# Abdominal aortic aneurysm in aged population

**DOI:** 10.18632/aging.101702

**Published:** 2018-12-06

**Authors:** Ryoko Umebayashi, Haruhito A. Uchida, Jun Wada

**Affiliations:** 1 Department of Chronic Kidney Disease and Cardiovascular Disease, Okayama University Graduate School of Medicine, Dentistry and Pharmaceutical Sciences, Okayama, Japan

**Keywords:** abdominal aortic aneurysms, cardiovascular disease, frailty, medical treatment, phosphodiesterase III

Abdominal aortic aneurysm (AAA) is pathologic dilation of the abdominal aorta and is often asymptomatic but has high susceptibility to rupture. The prevalence of AAA increases with age. Aortic aneurysm is the 13th leading cause of death in the United States and approximately 4,500 people per year die secondary to AAA rupture. The major risk factors for aortic aneurysm are age older than 65 years, male gender, family history, and smoking habit [1]. Other risk factors for AAA are common to those for other cardiovascular diseases except diabetes mellitus [2]. Since intact AAAs are typically asymptomatic, screening for AAA with ultrasound, especially in men aged 65 years and older, confers beneficial outcome [3].

Regarding treatment for aortic aneurysm, elective repair of AAA with open and endovascular surgical repairs are initially indicated in patients with large AAAs (> 5.5 cm) to prevent from aneurysms rupture [1]. Since AAA is common in elderly people, the risks of postoperative complications should be considered. Frailty is also common in elderly people. The prevalence of frailty increases with the coexisting cardiovascular diseases [4]. In addition to the classical risk factors for frailty, including age, female gender, cardiac failure, chronic obstructive pulmonary disease, renal impairment, cerebrovascular disease, peripheral artery disease, ischemic heart disease and diabetes mellitus, evaluation for frailty should be required for the risk assessment of AAA [5]. Endovascular repair is more optimal than open repair in patients with cardiopulmonary or other associated diseases. Furthermore, endovascular repair is useful option in patients who are unable to tolerate open surgery because of its minimally invasive therapy. However, endovascular therapy is unsuitable for patients with anatomical problems which increase highly graft complications rate and cause secondary interventions. Moreover, the morbidity and mortality associated with both techniques remain non-negligible [1]. Thus, the current surgical options still contain both the strengths and the weaknesses.

Upon such a limitation for surgical therapy, several medical options have been studied for management of small aortic aneurysm, regarding the ability to slow aneurysmal growth and to prevent from rupture. The aneurysmal growth and rupture are considered partially due to vascular inflammation, mechanical stress and matrix metalloprotease activities. The well management of blood pressure control reduces progression and rupture of AAAs, and β blockers were known to reduce perioperative mortality of AAAs, however the effect of anti-hypertensive drugs, including β blockers and renin-angiotensin system inhibitors, on AAA enlargement were not proven. Statins attenuated the development and growth of experimental AAA through its anti-inflammatory and anti-oxidative stress effects, however, several clinical studies failed to show their effect of AAA enlargement and ruptures. Doxycycline, a broad inhibitor of matrix-metalloproteinases, successfully demonstrated to slow AAA growth in small size human study. However, the efficacy on the AAA growth failed in phase II study. Cyclosporine, an inhibitor of cyclophilin A, also expected to suppress the development and progression of AAA by inhibiting inflammatory cell recruitment and matrix metalloprotease activities in animal studies. Short course administration of cyclosporine A have shown to stabilize the diameter of formed AAA in animal models, however, the effect and usage of cyclosporine on human AAA and is still investigating. The effect of the anti-platelet drugs on AAAs were not proven. Only a few of studies showed the preferable effects of anti-platelet drugs such as aspirin or P2Y12 inhibitors on the development and progression of AAAs and death from AAAs. We previously demonstrated that the potential ability of cilostazol, a PDE-III inhibitor, on AAA development thorough its anti-inflammatory effect [6]. Cilostazol is already used in clinical practice for patients with peripheral artery diseases and stroke. Since patient with AAA can often have many vascular complications including coronary artery disease, myocardial infarction and peripheral artery disease, our finding could provide a favorable option for the treatment in patients with AAA in clinical practice.

Aging related vascular diseases such as peripheral artery diseases and AAAs, will increase with aging of society, thus, the treatment options for these diseases will gain more attention in world wide.
